# Small RNA Profiling in Dengue Virus 2-Infected *Aedes* Mosquito Cells Reveals Viral piRNAs and Novel Host miRNAs

**DOI:** 10.1371/journal.pntd.0004452

**Published:** 2016-02-25

**Authors:** Pascal Miesen, Alasdair Ivens, Amy H. Buck, Ronald P. van Rij

**Affiliations:** 1 Department of Medical Microbiology, Radboud University Medical Center, Radboud Institute for Molecular Life Sciences, Nijmegen, The Netherlands; 2 Centre for Immunity, Infection & Evolution, University of Edinburgh, Edinburgh, United Kingdom; Colorado State University, UNITED STATES

## Abstract

In *Aedes* mosquitoes, infections with arthropod-borne viruses (arboviruses) trigger or modulate the expression of various classes of viral and host-derived small RNAs, including small interfering RNAs (siRNAs), PIWI interacting RNAs (piRNAs), and microRNAs (miRNAs). Viral siRNAs are at the core of the antiviral RNA interference machinery, one of the key pathways that limit virus replication in invertebrates. Besides siRNAs, *Aedes* mosquitoes and cells derived from these insects produce arbovirus-derived piRNAs, the best studied examples being viruses from the *Togaviridae* or *Bunyaviridae* families. Host miRNAs modulate the expression of a large number of genes and their levels may change in response to viral infections. In addition, some viruses, mostly with a DNA genome, express their own miRNAs to regulate host and viral gene expression. Here, we perform a comprehensive analysis of both viral and host-derived small RNAs in *Aedes aegypti* Aag2 cells infected with dengue virus 2 (DENV), a member of the *Flaviviridae* family. Aag2 cells are competent in producing all three types of small RNAs and provide a powerful tool to explore the crosstalk between arboviral infection and the distinct RNA silencing pathways. Interestingly, besides the well-characterized DENV-derived siRNAs, a specific population of viral piRNAs was identified in infected Aag2 cells. Knockdown of Piwi5, Ago3 and, to a lesser extent, Piwi6 results in reduction of vpiRNA levels, providing the first genetic evidence that *Aedes* PIWI proteins produce DENV-derived small RNAs. In contrast, we do not find convincing evidence for the production of virus-derived miRNAs. Neither do we find that host miRNA expression is strongly changed upon DENV2 infection. Finally, our deep-sequencing analyses detect 30 novel *Aedes* miRNAs, complementing the repertoire of regulatory small RNAs in this important vector species.

## Author Summary

Mosquitoes of the *Aedes* family transmit many important viruses, including dengue virus, between their vertebrate hosts. In the mosquito, the growth of these viruses is limited by the antiviral RNA interference pathway. Key to this pathway is a class of small non-coding RNAs known as small interfering RNAs (siRNAs). In addition, two related but distinct small RNA pathways known as the microRNA (miRNA) and the PIWI-interacting RNA (piRNA) pathway are implicated in regulating virus replication in mosquitoes. Thus, since small RNAs may critically influence the transmission of dengue virus, we set out to analyze the populations of viral and mosquito small RNAs that are produced in infected *Aedes* mosquito cells. We found that besides the well-known viral siRNAs, dengue virus-derived piRNAs were produced in these cells and we identified the PIWI proteins that these small RNAs rely on. In addition, we found that viral miRNAs were not expressed from the dengue virus genome and that the levels of mosquito miRNAs were barely changed upon infection. Finally, our data allowed for the identification of novel *Aedes* miRNAs, complementing the repertoire of these important regulatory RNAs in vector mosquitoes.

## Introduction

*Aedes* mosquitoes are essential vectors for the transmission of important arthropod-borne viruses (arboviruses), including dengue virus (DENV), yellow fever virus, and chikungunya virus [1]. While several of these arboviral infections cause disease in humans, virus replication generally does not lead to severe pathology in vector mosquitoes. Infected mosquitoes thus serve as a persistent reservoir for arboviruses in the wild and they may transmit these viruses to vertebrate hosts throughout their entire lives [2].

After ingestion in a mosquito’s blood meal, arboviruses need to overcome a number of anatomical and immunological barriers to reach sufficiently high titres in the saliva. Only then can transmission to a naive vertebrate host efficiently occur. One of the most important immune responses to arboviral infection is antiviral RNA interference (RNAi) [3–5]. This pathway is triggered by the presence of double stranded RNA (dsRNA), which is produced during the replication of RNA and DNA viruses [6,7]. The dsRNA is recognized and cleaved by the RNase-III enzyme Dicer-2 (Dcr2) into 21 nucleotide (nt) small interfering RNA duplexes (viral siRNA; vsiRNA) [8,9]. One of the siRNA strands is incorporated in Argonaute-2 (Ago2), the core protein of the RNA induced silencing complex (RISC) [10]. The siRNA-loaded RISC complex is guided to complementary viral RNA molecules and cleaves these target RNAs using the endonuclease (slicer) activity of Ago2 [11].

MicroRNAs (miRNAs) are a distinct class of small RNAs that are produced from genome-encoded stem loop-containing transcripts known as primary miRNA (pri-miRNAs). During the canonical miRNA biogenesis pathway, the stem loop structures, known as precursor miRNA (pre-miRNA), are released from the pri-miRNA by the microprocessor complex with at its core the RNase-III enzyme Drosha. After translocation into the cytoplasm, pre-miRNAs are cleaved by Dicer-1 (Dcr1) to produce a small RNA duplex comprised of the two mature miRNA strands. Usually, one of these strands is then preferentially incorporated into the Argonaute-1 (Ago1) containing miRNA-induced silencing complex (miRISC), whereas the other strand (the passenger or miRNA* strand) is usually discarded [12]. Loaded miRISC complexes are able to bind to specific target sites within mRNAs. This miRNA-mRNA interaction is initiated by nucleotide two to seven of the miRNA, the so-called seed sequence [13]. Stable binding of miRISC to an mRNA target, generally causes down-regulation of gene expression via translational inhibition and mRNA destabilization [14]. Importantly, infecting viruses can directly or indirectly, as a consequence of the immune response, reshape the host miRNA expression landscape. While quite a number of studies have reported on this matter in mammalian systems [15], little is known about virus-induced changes in miRNA levels in mosquito vectors. In *Aedes* mosquitoes, miRNA levels or modifications have been reported to be changed upon infections with DENV, West Nile virus, and chikungunya virus [16–20]. For most of these differentially expressed miRNAs, the biological relevance as well as the targeted mRNAs still await experimental validation.

Besides modulation of host miRNAs, some DNA and retroviruses encode their own miRNAs to regulate viral and host mRNAs [21]. The expression of miRNAs from cytoplasmic RNA viruses has been controversial. However, functional introduction of artificial miRNAs into the genomes of Sindbis virus (SINV) and tick-borne encephalitis virus provides evidence that miRNA production from cytoplasmic RNA viruses may in principle be possible [22,23]. Yet, the presence and biological relevance of miRNAs encoded in the genomes of flaviviruses such as DENV is still an issue of debate [24–26].

The third, most enigmatic class of small RNAs are PIWI interacting RNAs (piRNAs). These are processed from long RNA precursors that are transcribed from genomic loci known as piRNA clusters. In sharp contrast to siRNAs and miRNAs, their biogenesis into mature piRNAs is Dicer-independent. In *Drosophila*, piRNA maturation involves endonucleolytic cleavage of precursor transcripts by the Zucchini nuclease and the three PIWI proteins Piwi, Aubergine (Aub) and Argonaute-3 (Ago3) [27–30]. The primary function of the piRNA pathway in this model organism is the defence against transposable elements, mainly in germ-line tissues. Interestingly, piRNAs of viral origin (vpiRNA) have been found in somatic tissue of *Aedes* mosquitoes, suggesting that they contribute to the regulation of virus replication [31]. At present, vpiRNAs have been discovered upon infection with a number of Alphaviruses, Bunyaviruses and Flaviviruses, including DENV [31–39]. However, with the exception of SINV (Alphavirus), their molecular biogenesis has not been investigated [37].

Here, we make use of small RNA deep-sequencing in the siRNA, miRNA, and piRNA competent *Aedes aegypti* Aag2 cell line to investigate the production of small RNAs during DENV infection. We find that in addition to the well-characterized vsiRNAs, specific vpiRNAs are produced from DENV, which for their biogenesis in Aag2 cells rely on Piwi5 and Ago3 and, to a lesser extent, on Piwi6. We do not detect DENV-derived miRNAs, or prominent changes in host miRNA levels upon infection. Finally, we identify novel host miRNAs in our small RNA deep-sequencing libraries, complementing the currently annotated miRNA repertoire in *Aedes aegypti* vector mosquitoes.

## Materials and Methods

### Cells and viruses

Aag2 cells were cultured at 25°C in Leibovitz L-15 medium (Gibco) supplemented with 10% heat inactivated fetal calf serum (FCS; PAA), 2% tryptose phosphate broth solution (Sigma), 1x MEM non-essential amino acids (Gibco), and 50 U/ml penicillin and 50 μg/ml streptomycin (pen/strep; Gibco). U4.4 and C6/36 were kept in the same culture medium at 28°C. BHK-21 cells were cultured at 37°C, 5% CO_2_ in Dulbecco’s modified Eagles medium (DMEM) supplemented with 10% FCS and pen/strep. Stocks of DENV serotype 2 (DENV2), New Guinea C (NGC) and 16681 strains were grown on C6/36 cells and titred on BHK-15 cells as detailed in [40].

### Infection of Aag2 cells with DENV2

Aag2 cells were seeded one day prior to infection and infected with DENV2 at a multiplicity of infection (MOI) of 0.5 by directly adding the virus to the culture medium. Three days post infection the culture medium was removed and cells were harvested for RNA and protein isolation as detailed below.

### Western blot

For the detection of the DENV NS1 protein in samples used for small RNA deep-sequencing, 5% of the cells were harvested in 50 μl lysis buffer (50 mM Tris-HCl pH 7.8; 150 mM NaCl; 1 mM EDTA; 0.5% NP-40; 1x Protease inhibitor cocktail (Roche); 1 mM DTT). 12.5 μl of 5x Laemmli buffer was added to each sample, incubated at 95°C for 5 min, and 30μl of each sample was loaded on a 12.5% polyacrylamide gel. After gel electrophoresis, proteins were transferred to a nitrocellulose membrane (Bio-Rad) using a semi-dry blotting system (Bio-Rad). The membrane was blocked in 5% non-fat dry milk (Bio-Rad) in 0.1% Tween20 in PBS (PBS-T) for 30 min at room temperature. Mouse anti DENV NS1 antibody was kindly provided by Dr. Peter Mason [41]. The antibody was added to the membrane in a 1:1,000 dilution in 5% blocking buffer. After an incubation for 1.5 hours at room temperature, the membrane was washed three times in PBS-T. IRdye680 conjugated goat anti mouse antibody (1:15,000 dilution in PBS-T; Licor) was then added to the membrane and incubated at room temperature for 1.5 hours. After three washing steps, the membrane was imaged on an Odyssey infrared image system (Licor).

### dsRNA production and transfection of Aag2 cells

dsRNAs targeting PIWI/AGO transcripts or luciferase as a negative control were produced by *in vitro* transcription from T7-promoter flanked PCR products as detailed in [37]. Primers to produce T7-flanked PCR products are indicated in S1 Table.

For dsRNA transfection, 7.5x10^5^ Aag2 cells were seeded in one well of a 24-well plate. For each condition, three wells were plated. The following day, transfection mixes containing 300 μl non-supplemented L-15 medium, 450 ng dsRNA and 1.8 μl X-tremeGENE HP (Roche) were prepared according to the manufacturer’s recommendations. 100 μl of the mix was added dropwise to one well. After 2–3 hours the medium was replaced with fully supplemented L-15 medium. 48 hours later, the transfection was repeated to enhance knockdown efficiencies. Where indicated, the cells were infected with DENV2 which was added to the L-15 medium used to replace the transfection medium as specified above.

### RNA isolation

Aag2 cells were lysed in Isol-RNA Lysis reagent (5 PRIME) as described in the manufacturer’s instructions. Briefly, 200 μl of chloroform was added to 1 ml of Lysis reagent and mixed well. After centrifugation, the aqueous phase was collected and total RNA was purified using isopropanol precipitation. RNA was quantified on a Nanodrop spectrophotometer and RNA integrity was checked by ethidium bromide staining of ribosomal RNA bands after agarose gel electrophoresis.

### DNaseI treatment, reverse transcription and (quantitative) PCR

For RT-(q)PCR, 1 μg of total RNA was DNaseI (Ambion) treated according to the manufacturer’s instructions. The RNA was subsequently reverse transcribed in a 20 μl reaction using the Taqman reverse transcription kit (Applied Biosystems). Complementary DNA (cDNA) was diluted 5–10 times before PCR amplification. Endpoint PCR was performed using Thermoperfect DNA Polymerase. Quantitative PCR (qPCR) analysis was performed using the GoTaq qPCR SYBR mastermix (Promega) on a LightCycler 480 instrument (Roche). The relative changes in gene expression were calculated using the ΔΔCt method [42] using lysosomal aspartic protease (LAP) as an internal normalization control. Sequences of the PCR primers are indicated in S1 Table.

### β-elimination

Sodium periodate (NaIO_4_) oxidation and β-elimination of total RNA was performed as described previously [43]. Total RNA (10 μg in 47.5 μl nuclease-free water) was mixed with 12.5 μl 200 mM NaIO_4_ and 40 μl 5x borate buffer. As a control, RNA was treated with water instead of NaIO_4_. The reaction was incubated at room temperature for 30 min and 10 μl glycerol was added to the reaction. The reaction was incubated for another 10 min before 10 μl of 500 mM sodium hydroxide (NaOH) was added to induce β-elimination. The reaction was incubated at 45°C for 90 min. After these treatments, total RNA was purified by ethanol precipitation in the presence of 300 mM NaCl and 5 μg of glycogen. Electrophoretic mobility of *Aedes aegypti* miR-2940-3p and DENV2 piRNAs was then analyzed by small RNA northern blotting as detailed below.

### Small RNA northern blotting

Small RNA northern blot was performed as described in [44]. Briefly, total RNA was size-separated on 0.5x TBE, 7 M Urea, 15% Polyacrylamide gels, transferred to Hybond NX nylon membranes (Amersham), and cross-linked using 1-ethyl-3-(3-dimethylaminopropyl) carbodiimide (EDC; Sigma). Individual small RNAs were detected with DNA oligonucleotides that were 5’ end-labelled with [^32^P] γ-adenosine-triphosphate (Perking Elmer) using T4 Polynucleotide kinase (Roche). Hybridization to the oligo-probes was performed overnight at 42°C in Ultrahyb Oligo hybridization buffer (Ambion). Membranes were then washed three times at 42°C in 0.1% SDS with decreasing concentrations of SSC (2x, 1x, 0.1x). Membranes were exposed to X-ray films (Carestream) or Phosphorimager screens (BioRad). Sequences of DNA oligonucleotide probes are indicated in S1 Table. Quantification of northern blot panels was performed using ImageJ software. Bands were defined using the rectangular selection tool and the pixel density (area under the curve) was measured and normalized to uninfected dsLuc samples.

### Preparation of small RNA libraries

Small RNA libraries were prepared as described previously [37,45]. Briefly, three 25 cm^2^ flasks of Aag2 cells were infected in parallel with DENV2 NGC. Three additional flasks were left uninfected. Total RNA was then isolated from these six flasks as specified above and 30 μg of total RNA was size separated on a 15% Polyacrylamide, 7 M urea, 0.5x TBE gel. Subsequently, the small RNAs in the size range from 18 nt to 33 nt were excised from gel using radioactively-labelled RNA oligos, loaded in the adjacent lanes of the gel, as rulers. The gel was crushed and the small RNAs were eluted in 300 mM sodium acetate overnight at 4°C under constant rotation. The RNA was recovered from the elution buffer using ethanol precipitation and eluted in 10 μl of nuclease-free water. 5 μl of the sample was directly used as input for small RNA deep-sequencing library preparation using the TruSeq small RNA library preparation kit (Illumina) following the manufacturer’s recommendations. The RNA was ligated to 3’ and 5’ adapters, reverse transcribed, and PCR amplified. The small RNA libraries were then size-purified from 1x TBE, 6% polyacrylamide gel using overnight elution in 300 mM sodium acetate followed by ethanol precipitation. The individual small RNA libraries were pooled and sequenced on a single sequencing lane on a HighSeq2500 by Baseclear (Leiden, The Netherlands).

### Viral small RNA profiling

FASTQ sequence reads were generated using the Casava pipeline (v.1.8.3) and initial quality analysis was performed using the Illumina Chastity filter and an in-house filtering protocol by Baseclear. Subsequent quality assessment was based on the FASTQC quality control tool (v.1.10.0). The individual small RNA sequencing libraries were separated based on the TruSeq indices (no. 1 to 6) that were introduced during PCR amplification. The individual libraries were subsequently analyzed using the Galaxy bioinformatics tool shed [46,47]. For the analysis of viral small RNAs, reads were mapped to the DENV2 NGC genome (GenBank accession: KM204118.1) using Bowtie (v.1.1.2) [48]. Size profiles were obtained from all reads that align to this reference sequence with a maximum of one mismatch. The genome distribution of 21 nt siRNAs, 22–24 nt small RNAs, or 25–30 nt piRNAs was obtained by plotting the number of 5’ ends of these reads at each position of the genome. For the pileup plots of UTR-derived miRNA-like small RNAs, the 22–24 nt small RNAs were selected from the initial FASTQ files and mapped to the DENV-NGC genome. From the resulting SAM files the reads mapping to the (+) strand of the virus genome were selected and used as input for the ‘Generate pileup from BAM dataset’ tool (v.1.1.2). The values at the nucleotide positions of the DENV2 5’UTR (1–96) and 3’ UTR (10273–10723) were selected for display.

Re-analysis of the data published by Hess et al. [36] was performed on the dataset with the accession number SRR921363. The SOLiD-formatted dataset was groomed to fit the requirements for manipulation in Galaxy. After adapter clipping, reads were mapped to the DENV2-JAM1409 genome (GenBank accession: M20558) using Bowtie2 [49]. Size and genome profiles were obtained as described above. Unless specified differently, all small RNA read counts were normalized against the size of the corresponding sequencing library and are expressed as ‘% of the library’ (i.e. reads per hundred).

### miRNA analysis and prediction

Analysis of miRNA expression levels was performed using the miRDeep2 tool. Raw data were assessed for quality using FASTQC. Subsequently, adapters were removed from the raw reads, and the reads were quality trimmed using cutadapt software (http://dx.doi.org/10.14806/ej.17.1.200) with parameters -O 6 -m 17 -n 5 -q 20. Within each library, the resulting reads were collapsed to generate a non-redundant set of FASTA sequences, subsequently processed to the format required for miRNA prediction with miRDeep2 software [50]. Collapsed reads longer than 17 nucleotides were aligned to the *Aedes* genome (assembly AaegL3, downloaded from vectorbase) and the DENV2 NGC genome using the mapper.pl component of miRDeep2 (parameters: -o 20 -l 19 -r 100 -c). The resulting outputs were parsed to remove alignments that were not full length and perfect match (FLPM). miRDeep2 predictions were generated from the FLPM aligned sequences, with miRBase v21 Arthropoda mature miRNA and pre-miRNA sequences as templates [51]. The ‘miRNAs_expressed’ output from miRDeep2, which comprises tallies for each known miRNA in each sample, was further processed in the R/Bioconductor environment. Briefly, miRNA read counts were normalized to the number of *Aedes*-specific genome reads within each sample group, using the lowest number of reads aligning as the baseline. Subsequently, the counts were converted to abundances within each sample, converted to log2 equivalent counts, and all samples quantile normalized prior to linear model fitting with the limma package [52]. MiRNA predictions from the miRDeep2 output were manually curated using the following criteria: i) high-confidence miRNA predictions have reads mapping to both a predicted mature and star sequence, ii) the mature sequences have a homogenous 5’ end (80% of reads start at same position), and iii) miRNA-miRNA* duplex should resemble a Dicer product on a genomically-encoded hairpin, having a two nt (+/-1 nt) overhang at the 3’ end. miRNA predictions supported by >1000 reads as well as miRNA predictions with a seed match to known insect miRNAs were also kept. To be retained, these predictions required a homogenous 5’ end, but did not require the presence of reads mapping to the expected star strand. miRNA names and accession numbers were assigned by the miRBase repository.

All deep sequencing libraries have been submitted to NCBI Sequence Read Archive under the accession number SRA303329. All source data are available in S1 Dataset.

## Results

### DENV2-derived small RNAs in infected Aag2 cells

**Fig 1:**
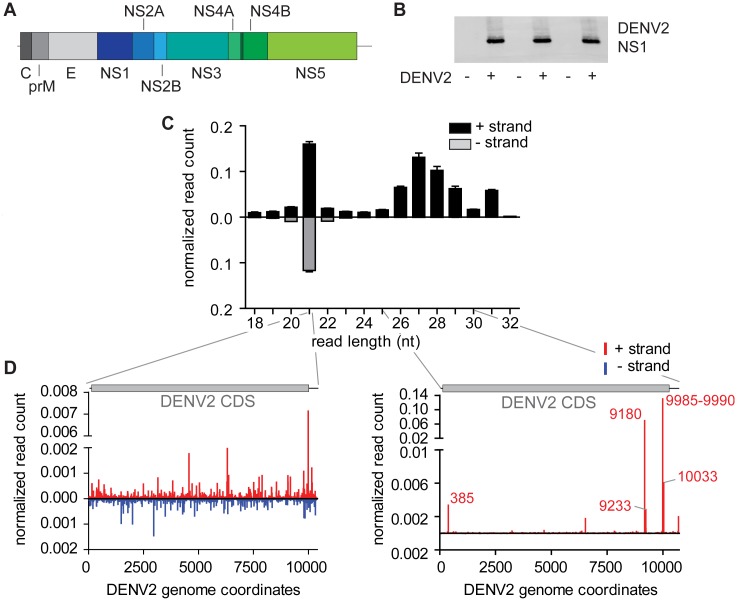
Small RNA production in DENV infected Aag2 cells. **(A)** Schematic representation of the DENV NGC genome (accession KM204118; 10723 bp). Structural proteins are indicated in grey scale, non-structural proteins are displayed in blue to green scale. **(B)** Western blot against the DENV2 NS1 protein in the three infected and uninfected samples used for small RNA library preparation. **(C)** Size profile of small RNAs mapping to the DENV2 genome with a maximum of one mismatch. Black bars represent reads mapping to the (+) strand of the genome, grey bars depict reads from the (-) strand. The read counts have been normalized to the size of the small RNA library and the mean +/- standard error of the mean (SEM) is presented (n = 3). **(D)** Distribution of 21 nt vsiRNAs (left panel) or 25–30 nt small RNAs (right panel) across the DENV genome. Reads from the (+) and (-) strands are depicted in red and blue, respectively. The read counts have been normalized as described in C, the mean read count of the three libraries is shown. Numbers in red indicate genome positions of the vpiRNA spikes.

DENV is a positive (+) strand RNA virus belonging to the *Flavivirus* genus in the *Flaviviridae* family. Its RNA genome is approximately 10.7 kilobases in size and encodes a single polypeptide that is processed by proteolytic cleavage events into three structural proteins and seven non-structural proteins (Fig 1A). Since various classes of small RNAs have been implicated in modulating DENV infections in its mosquito vectors, we aimed to characterize the repertoire of virus and host-derived small RNAs in *Aedes aegypti* Aag2 cells. To this end, we prepared three independent small RNA deep-sequencing libraries from uninfected and DENV2 (NGC strain) infected cells, each. The efficiency of the three infections was comparable as assessed by western blot for the DENV2 NS1 protein (Fig 1B). As expected, DENV2-derived viral small RNAs showed a clear population of 21 nt vsiRNAs mapping to both the viral positive (+) strand and the negative (-) strand in roughly equal numbers (Fig 1C). Interestingly, besides siRNAs, a second population of viral small RNAs was produced that resembled vpiRNAs. These were 25–30 nt in length and almost exclusively derived from the viral (+) strand (Fig 1C). In contrast to vsiRNA, which were distributed along the entire length of the viral genome, these putative vpiRNAs were produced only from few specific positions (Fig 1D). In fact, 85% of all the 25–30 nt reads were derived from four individual vpiRNA sequences, present in the NS5 gene at positions 9180 and 9985, 9989 and 9990 of the DENV2 NGC genome. To test whether these small RNA profiles reflect those from adult mosquitoes, we re-analyzed deep sequencing data from DENV2 infected *Aedes aegypti* mosquitoes published by Hess *et al*. [36]. We analyzed the 9 days post infection sample, which showed the highest number of viral siRNAs and piRNAs. Whereas normalized vsiRNA levels were only 2.2-fold lower in these libraries than in our Aag2 data, vpiRNAs were about forty times lower. Yet, the viral small RNA profiles were strikingly similar, with 21 nt reads being scattered throughout the entire viral genome and piRNA-sized reads being predominantly produced from few positions located towards the 3’ end of the viral genome (S1 Fig). These data suggest that similar mechanisms might produce viral piRNAs in Aag2 cells and adult mosquitoes.

### vpiRNA production from DENV2 RNA

**Fig 2:**
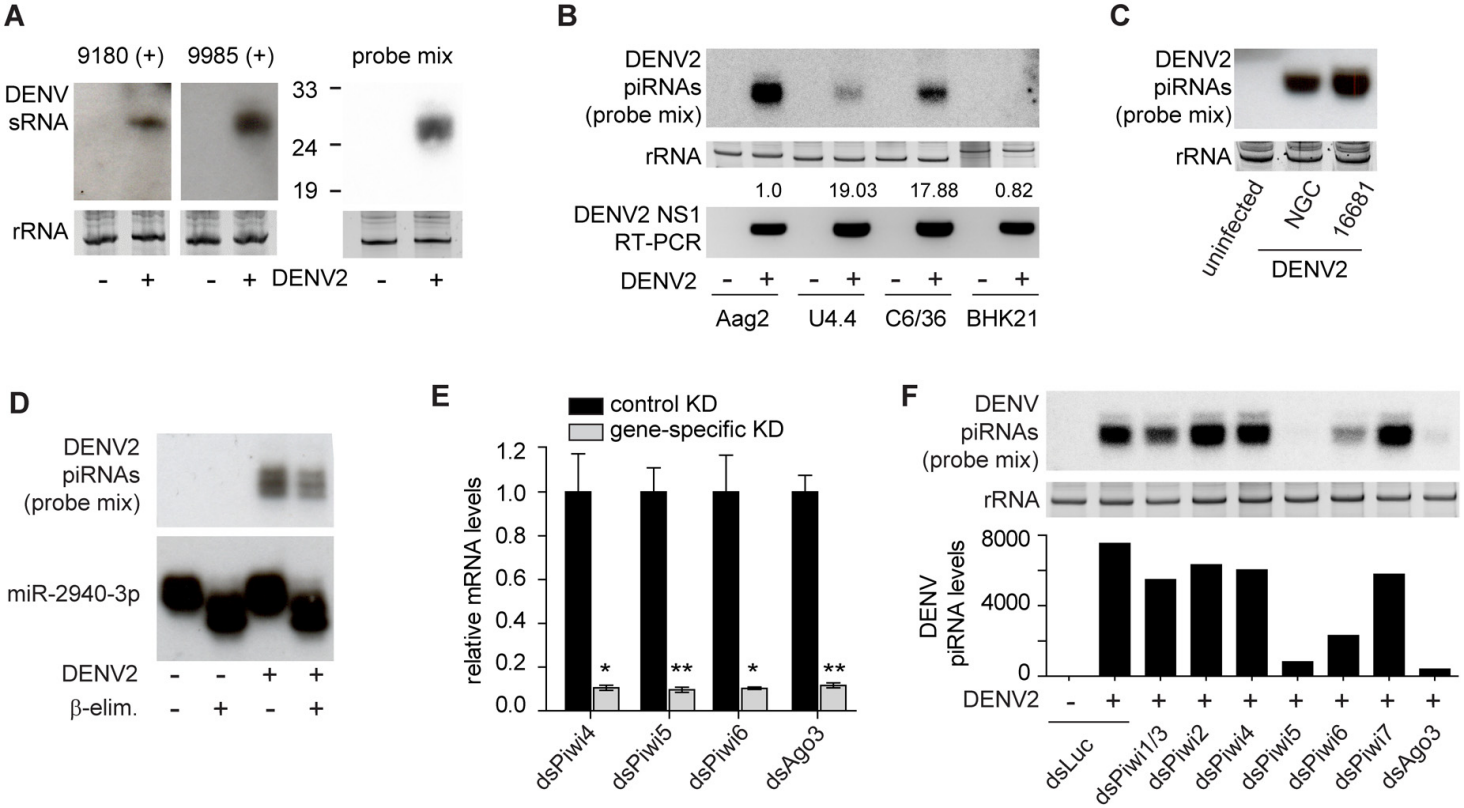
vpiRNA production in Aag2 cells. **(A)** Northern blot of highly abundant vpiRNAs. Two individual DNA oligonucleotide probes (left panels), or a combination of these probes (right panel) were used to detect the small RNAs. The combination of probes was used in all subsequent small RNA blots. **(B)** Upper panel: Small RNA northern blot of vpiRNAs in the indicated cell lines after infection with DENV2. Lower panel: RT-PCR for DENV genomic RNA in the same samples used for the northern blot. Numbers on top indicate DENV genomic RNA levels (relative to Aag2 cells) as determined by RT-qPCR (n = 1). **(C)** Northern blot of DENV2 piRNAs in Aag2 cells infected with the DENV2 NGC or 16681 strain, both at an MOI of 0.5. **(D)** Northern blot of DENV2 piRNAs and *Aedes* miR-2940-3p in uninfected or DENV2 infected Aag2 cells. Where indicated, total RNA was subjected to β-elimination. **(E)** RT-qPCR for the indicated PIWI proteins after gene-specific knockdown (KD) in Aag2 cells normalized to a control KD (dsLuc). Bars represent the mean of three experiments +/- SEM. Statistical significance was determined using two tailed, unpaired student t-test. * p<0.05; **p<0.01. **(F)** Upper panel: Small RNA northern blot of vpiRNAs upon KD of the indicated PIWI proteins. RNA samples analyzed in E were pooled for this blot. Lower panel: Quantification of two independent blots including the one shown in the upper panel using ImageJ software. For the other blot, see S1 Dataset. Ethidium bromide staining of ribosomal RNA was used as loading control in panel A, B, C, and F.

To exclude the possibility that the piRNA-like molecules are sequencing artefacts and to characterize this small RNA population in more detail, we performed small RNA northern blotting for the highly-abundant small RNAs starting at DENV2 genome positions 9180 or 9985–9990. Indeed, small RNAs in the expected size range could readily be detected specifically in DENV2 infected Aag2 cells (Fig 2A). In addition, these sequences were also present in DENV2-infected U4.4 and C6/36 cells derived from *Aedes albopictus* mosquitoes, which we have previously shown to be competent in producing SINV-derived vpiRNAs [32,37] (Fig 2B). The levels of vpiRNAs did not correlate with the expression of viral genomic RNA. Whereas viral RNA levels were roughly eighteen to nineteen fold higher in U4.4 and C6/36 cells than in Aag2 cells, vpiRNAs were most abundant in Aag2 cells (Fig 2B). These data suggest that the composition of host factors required for their biogenesis is most favourable in Aag2 cells. As expected, mammalian BHK-21 cells, which lack an active piRNA pathway, did not produce vpiRNAs (Fig 2B). To exclude that viral piRNA production is an artefact of the use of the specific DENV2 strain, we analyzed piRNA accumulation in Aag2 cells infected with either the DENV NGC or DENV 16681 strain. Small RNA northern blotting revealed that infection with either strain resulted in the production of those viral piRNA sequences that we had found by deep-sequencing (Fig 2C). Next, we aimed to test whether DENV2-derived piRNAs are methylated at their 3’ end. To this end, we performed sodium periodate oxidation followed by beta-elimination, which reveals modifications of the 3’ terminal nucleotide of RNA molecules [43]. Unmodified small RNAs, such as animal miRNAs, are susceptible to this treatment and will be shortened by one nucleoside resulting in increased electrophoretic mobility. In contrast to miRNAs, piRNAs are protected against this treatment by methylation of the 2’OH on the ribose of the 3’ terminal nucleotide. Indeed, beta-elimination resulted in increased electric mobility of miR-2940-3p. Yet, the migration of DENV2 piRNA bands was not affected by beta-elimination, indicating that their 3’ terminal nucleotides are modified, most likely methylated (Fig 2D). Since piRNA methylation occurs after loading into PIWI protein complexes, these data suggest that the identified DENV2 piRNAs are mature piRNAs associated with a PIWI protein.

To identify which PIWI proteins are required for the biogenesis of DENV2 piRNAs in Aag2 cells, we individually knocked down expression of all the eight *Aedes* PIWI proteins and analyzed the production of vpiRNAs by northern blot. We confirmed knockdown efficiency of roughly 90% for the four PIWI proteins that are detectable by RT-qPCR in Aag2 cells (i.e. Piwi4, Piwi5, Piwi6, Ago3; Fig 2E). Expression levels of Piwi1-3 and Piwi7 were too low to allow reliable quantification. DENV2 piRNAs were almost undetectable upon knockdown of Piwi5 and Ago3 and clearly reduced upon knockdown of Piwi6 (Fig 2F). These data confirm that the 25–30 nt population of DENV2-derived small RNAs are *bona fide* piRNAs that require host PIWI proteins for their biogenesis. To test whether knockdown of PIWI expression results in enhanced DENV2 replication, we performed RT-qPCR to compare viral RNA levels in the different knockdown conditions. We found that none of the knockdowns resulted in a significant change in viral RNA levels (S2A Fig). Yet, also knockdown of the well-established antiviral factor Ago2 [53] only resulted in a minor, statistically not-significant, increase of viral RNA replication although knockdown efficiency was higher than 90% (S2B Fig). This suggests that, in our hands, knockdown of small silencing pathway components in Aag2 cells is not suited to uncover robust antiviral activity against DENV2.

### DENV2 miRNA-like small RNAs are not expressed in Aag2 cells

**Fig 3:**
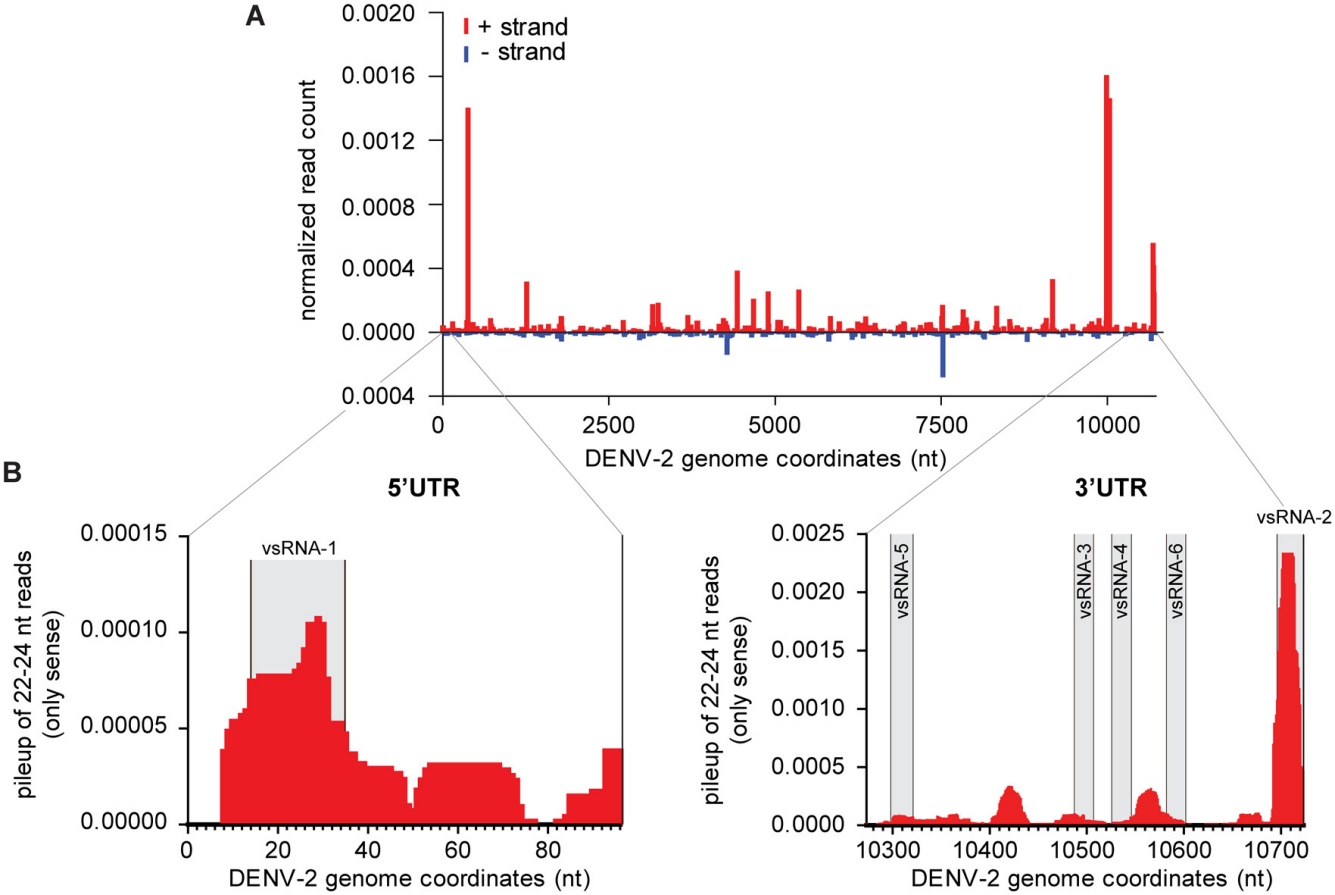
DENV miRNA-like small RNAs are not produced in infected Aag2 cells. **(A)** Distribution of 22–24 nt RNA reads across the DENV genome. Red bars indicate the number of 5’ ends of small RNAs that map to the (+) strand of the genome, blue bars represent the small RNAs mapping to the (-) strand. Read counts were normalized to the size of the corresponding library and the mean of the three libraries is plotted. **(B)** Pile-up of 22–24 nt small RNAs mapping to the (+) strand of DENV2 5’ (left panel) and 3’ UTRs (right panel), respectively. The mean of the three libraries is shown. Grey shadings highlight the boundaries of the mature DENV2 vsRNA sequences as reported in [24].

The significance of viral miRNA production from DENV genomic RNA is heavily debated [24–26,54]. To investigate whether viral miRNA-like molecules are produced in DENV2 infected Aag2 cells, we filtered 22–24 nt small RNA reads that map to the DENV2 genome with a maximum of one mismatch. In general, the number of 22 to 24 nt reads was rather low (~5% of all DENV2 mapping reads) when compared to 21 nt siRNAs (~28%) and 25-30nt piRNAs (~40%). Furthermore, there were only four outstanding peaks that gave rise to a somewhat higher number of small RNAs (Fig 3A). All of them coincided with the position of a vpiRNA peaks (Fig 1D), suggesting that these small RNAs were by-products of vpiRNA production. Parallel analysis of the virus-derived reads using miRDeep2 did not identify convincing miRNA-like candidates: some reads mapped to two predicted hairpin sequences (genome positions: 9542 (+) strand; 4888 (-) strand), however the mapping patterns showed heterogeneity of the 5’ start sites and did not suggest Dicer processing (S3A Fig).

Recently, eight miRNA-like small RNAs were computationally predicted based on hairpin structures in the DENV2 genome, but they were not experimentally validated [54]. We specifically looked for small RNA reads in our sequencing data mapping in the proximity of these predicted viral miRNAs, allowing a margin of 3nt around the start site. For each of the predicted miRNAs, we identified only very few (<20) reads in the combined set of DENV2-infected small RNA libraries (total of >3.7·10^7^ reads of which >3.6·10^5^ are DENV specific). In another publication, several ‘miRNA-like’ RNAs (termed vsRNA-1 to 6) were proposed to be produced from the 5’ and 3’ UTRs of the DENV2 RNA, based on the analysis of small RNA sequencing data [24]. The DENV UTRs are indeed prone to form RNA structures and hairpins, which were suggested to be processed by the miRNA machinery into specific small RNA species [24]. We specifically looked for the proposed vsRNA sequences in our dataset but could only identify a small RNA population that resembled vsRNA-2 located at the terminal hairpin of the DENV2 genome (Fig 3B). However, small RNAs mapping in that region showed a broad size distribution with the majority ranging in size from 26 to 28 nt, arguing against vsRNA-2 small RNAs being *bona fide* Dicer products (S3B and S3C Fig). These data suggest that the proposed DENV2 vsRNAs are not an abundant class of small RNAs.

### Host miRNA levels are only mildly affected by DENV2 infection

**Fig 4:**
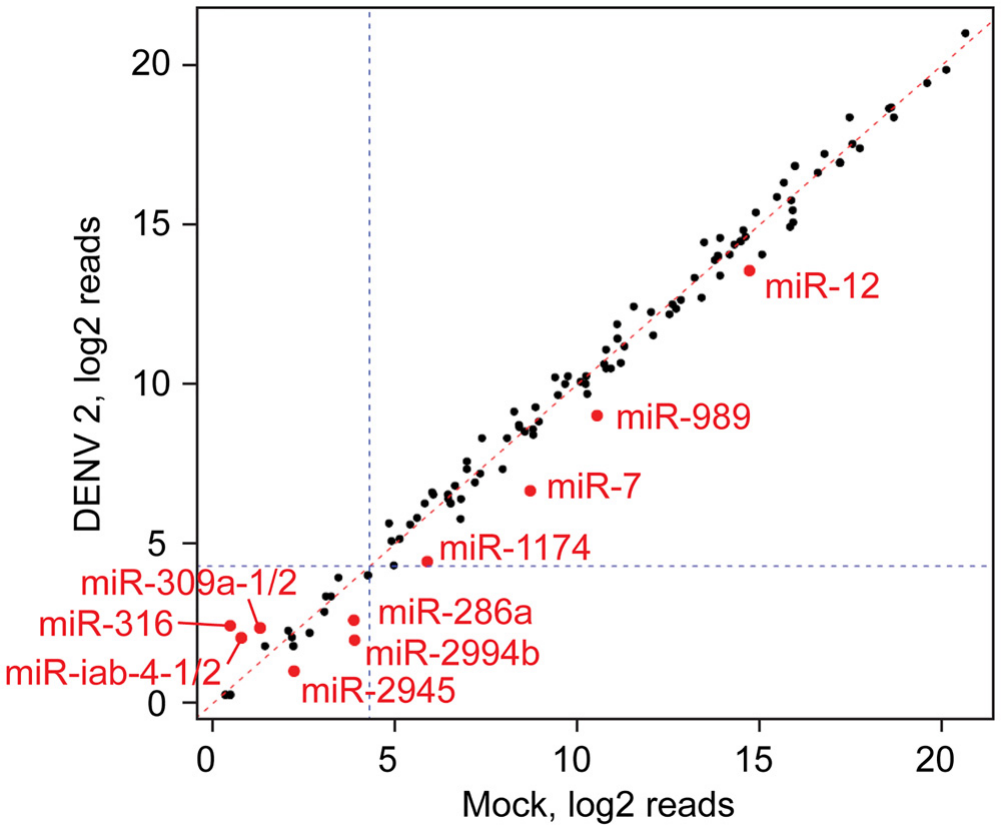
Host miRNA levels are not affected by DENV2 infection. Correlation of expression levels (log2-transformed) of known *Aedes aegypti* miRNAs in uninfected (x-axis) and DENV2 infected (y-axis) Aag2 cells. Mean expression of miRNAs across the three small RNA library replicates was determined using miRDeep2. Highlighted miRNAs are changed >2 fold. After p-value correction for multiple testing, no change in miRNA expression is statistically significant.

Host miRNAs function as key regulators of gene expression and changes in miRNA expression have been reported during virus infections in various animal hosts, including mosquitoes. To assess host miRNA expression in response to DENV2 infection, we made use of the miRDeep2 toolkit to quantify miRNAs in the uninfected and DENV2-infected Aag2 small RNA libraries. DENV2 infection caused only minimal changes in miRNA levels (Fig 4). The expression of three and seven miRNAs was changed more than 2-fold up or down, respectively in response to DENV infection. Yet, the majority of differentially regulated miRNAs, including all up-regulated miRNAs, were poorly expressed (mean expression levels below twenty reads) making it hard to discriminate these expression changes from experimental noise due to low read counts. Collectively, these data suggest that miRNA expression in Aag2 cells is not heavily affected by DENV infection.

### Novel *Aedes aegypti* miRNAs

**Fig 5:**
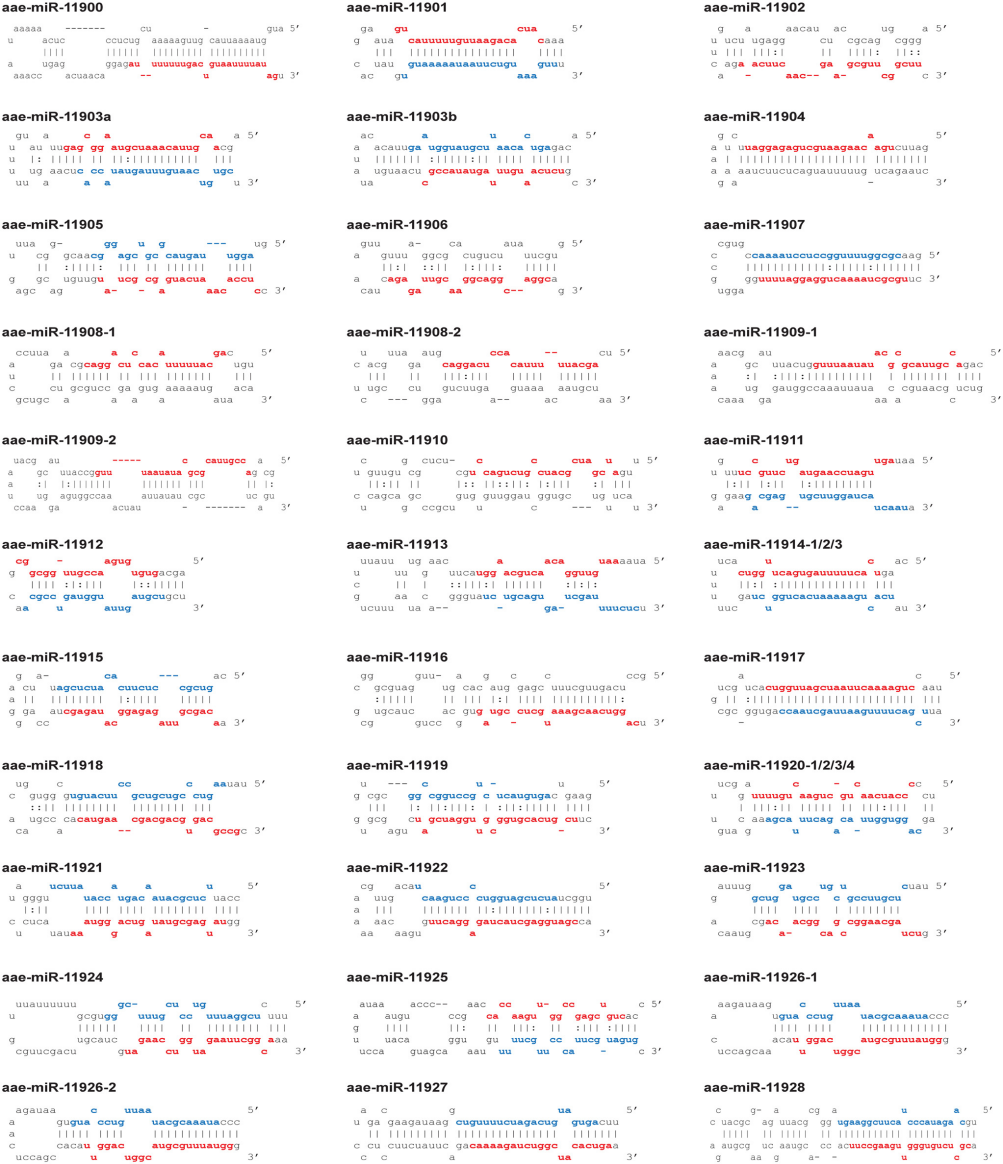
Novel *Aedes* aegypti pre-miRNAs. Hairpin structures of novel pre-miRNAs, as predicted using the RNAfold algorithm. Red letters indicate the position of the predicted mature miRNA sequence. Blue letters indicate the miRNA* strand. No miR* strand was found for ten predictions, representing nine eight miRNAs; these predictions are supported either by a seed-sequence known in insects or by high read counts. The mature miRNA sequences of aae-miR11908, aae-miR-11909 and aae-miR-11926 map to multiple different hairpins in the *Aedes* genome. For aae-miR-11914 and aae-miR-11920, the entire hairpin is encoded at multiple locations in the *Aedes* genome, as specified in Table 1.

The most recent version of miRBase (version 21) contains 101 *Aedes aegypti* miRNAs, which is considerably less than for other insect species including *Drosophila melanogaster* (fruitfly; 256 miRNAs), *Apis mellifera* (honey bee; 254 miRNAs) or *Bombyx mori* (silkworm; 487 miRNAs). We therefore suspected that the repertoire of published mosquito miRNAs is not yet complete and we set out to identify novel *Aedes aegypti* miRNAs in our small RNA sequencing data using miRDeep2. We obtained a list of 399 miRNA predictions (S2 Table), 73 of which were known miRNAs annotated in miRBase [51,55,56]. We also confirmed 16 miRNAs that were recently reported by Akbari *et al*. or Hu *et al*., but were not yet available in miRBase [57,58] (S4 Fig). The remaining 310 predictions of miRNA hairpins were manually inspected for novel miRNAs using a similar approach as described in [59,60], based on three criteria. First, only hairpin predictions that were supported by at least 1000 mature miRNA reads or those with at least one predicted miRNA* strand were retained. If a miRNA had an identical seed to a known insect miRNA, it was also retained irrespectively of read count or the presence of a miRNA*. In total 140 predictions met these criteria. Second, mature miRNAs were inspected for a homogeneous 5’ end of the supporting small RNA reads, defined as having at least 80% of the miRNA reads starting at the same nucleotide. 68 miRNA hairpin predictions met this requirement. Third, the predicted miRNA duplex was checked to resemble a (near) perfect Dicer product, defined as mapping to the stem of a stem-loop structure with one, two or three nucleotide overhangs at the 3’ end. Using this approach, we identified 31 unique mature miRNAs sequences mapping to 39 predicted miRNA hairpins. Nine of the 31 mature miRNA predictions did not have reads mapping to the star strand, but were supported by a homogeneous 5’ end in combination with a seed-match to an insect miRNA or having >1000 reads (Table 1, Fig 5). Further inspection identified one of these predictions to be derived from a tRNA which has therefore been removed from the list of predicted miRNAs.

**Table 1:** Novel *Aedes aegypti* miRNAs.

Name	ID	Position of the hairpin(s)	Mature miRNA mean read count*		Sequence of the mature miRNA	length
			Uninf.	DENV2		
aae-miR-11900†	MI0037941	supercont1.245 [–]: 1561005–1561086	79.7	31.9	auuuuuuugacuguaauuuuauag	24
aae-miR-11901	MI0037942	supercont1.885 [+]: 342921–342978	6.5	4.1	caucacagaauuguuuuuacug	22
aae-miR-11902†	MI0037943	supercont1.71 [+]: 1610981–1611037	2.6	5.0	aacuucaacgaagcguucggcuu	23
aae-miR-11903a	MI0037944	supercont1.484 [–]: 464269–464333	2.4	1.8	aacguuacaaaucguaaggcgag	23
aae-miR-11904†	MI0037945	supercont1.379 [+]: 580338–580393	0.6	1.4	ugaacaagaaugcugagaggau	22
aae-miR-11905	MI0037946	supercont1.14 [–]: 906240–906306	1.3	0.6	uaucgcgaguacuaaacaccuc	22
aae-miR-11906†	MI0037947	supercont1.517 [+]: 749300–749352	1.0	0.7	agagauugcaaggcaggcaggc	22
aae-miR-11907	MI0037948	supercont1.160 [+]: 1335655–1335712	0.5	0.4	uuuuaggaggucaaaaucgcgu	22
aae-miR-11908-1/2†	MI0037949	supercont1.70 [–]: 443560–443626	0.7	0.4	agcauuuuuacaccucaggac	21
	MI0037950	supercont1.369 [–]: 1123285–1123351				
aae-miR-11909-1/2†	MI0037951	supercont1.8 [–]:934642–934718	0.5	0.3	accguuacgcgcauauaauuug	22
	MI0037952	supercont1.41 [–]: 1367904–1367977				
aae-miR-11910†	MI0037953	supercont1.5 [+]: 2047651–2047726	0.3	0.3	aucgaucgcauccgucugaccu	22
aae-miR-11911	MI0037954	supercont1.89 [+]: 2726022–2726075	2.1	2.2	aguugauccaaguagucuugccu	23
aae-miR-11912	MI0037955	supercont1.48 [–]: 2566398–2566441	2.6	3.1	gugugugaaccguuggcggc	20
aae-miR-11913	MI0037956	supercont1.1336 [+]: 85845–85922	2.0	2.9	aauguuggacaacugcaaggu	21
aae-miR-11914-1/2/3	MI0037957	supercont1.1893 [–]: 19601–19653	1.5	2.3	ucacuuuuuagugacuugguc	21
	MI0037958	supercont1.224 [+]: 1335173–1335225				
	MI0037959	supercont1.222 [–]: 1778752–1778804				
aae-miR-11903b	MI0037960	supercont1.701 [+]: 403301–403361	1.8	0.7	cgccauaugauuuguaacucu	21
aae-miR-11915	MI0037961	supercont1.123 [–]: 1296986–1297043	0.5	0.6	cgagauacggagagauugcgaca	23
aae-miR-11916†	MI0037962	supercont1.916 [–]: 285767–285846	22.0	28.1	gaugccucguaaagcaacuggac	23
aae-miR-11917	MI0037963	supercont1.135 [+]: 1523348–1523407	3.1	4.0	cugaaaacuuaaucgauugguc	22
aae-miR-11918	MI0037964	supercont1. 151[–]: 1291075–1291138	3.1	2.4	caugaacgacgacgugacgccg	22
aae-miR-11919	MI0037965	supercont1.235 [–]: 964754–964810	3.3	3.0	uagcuagguugcggugcacugcu	23
aae-miR-11920-1/2/3/4	MI0037966	supercont1.441 [–]: 390672–390730	0.9	2.0	cccaucaacugcugaacuguuuu	23
	MI0037967	supercont1.49 [+]: 557677–557735				
	MI0037968	supercont1.16 [+]: 2615847–2615905				
	MI0037969	supercont1. 496 [+]: 473980–474038				
aae-miR-11921	MI0037970	supercont1.339 [+]: 1278980–1279041	2.5	2.0	aaaugggacugauaugcgaguau	23
aae-miR-11922	MI0037971	supercont1.551 [+]: 468137–468200	1.6	1.7	uucaggagaucaucgagguagc	22
aae-miR-11923	MI0037972	supercont1.220 [–]: 213093–213151	1.6	2.1	acaacggcagccggaacgaucu	22
aae-miR-11924	MI0037973	supercont1.657 [–]: 493003–493076	1.2	0.6	uagaaccugguagaauucggca	22
aae-miR-11925	MI0037974	supercont1.164 [–]: 201696–201781	0.4	0.9	cugucgagccgguugaaccac	21
aae-miR-11926-1/2	MI0037975	supercont1.607 [–]: 541794–541859	0.7	0.6	uuggacuggcaugcguuuaugg	22
	MI0037976	supercont1.21 [–]: 2803780–2803844				
aae-miR-11927	MI0037977	supercont1.97 [–]: 2388289–2388362	0.5	0.5	caaaagaucuggcuacacuga	21
aae-miR-11928	MI0037978	supercont1.215 [–]: 177863–177948	0.3	0.2	uuccgaaguugggugucucgc	21

miRNA predictions above the first thick line have a seed sequence that is present in an insect species, miRNAs between the two thick lines have a seed sequence that is present in a metazoan species, and miRNAs below the second thick line have seed sequences that are not present in any metazoan miRNA.

* The mean read count is normalized to the size of the corresponding small RNA sequencing library and presented as reads per million.

^†^ miRNA prediction is supported by a seed match to known insect miRNAs or >1000 reads (equivalent to appr. 80 rpm), but not by the presence of a star strand.

### miRNA offset RNAs in Aag2 cells

**Fig 6:**
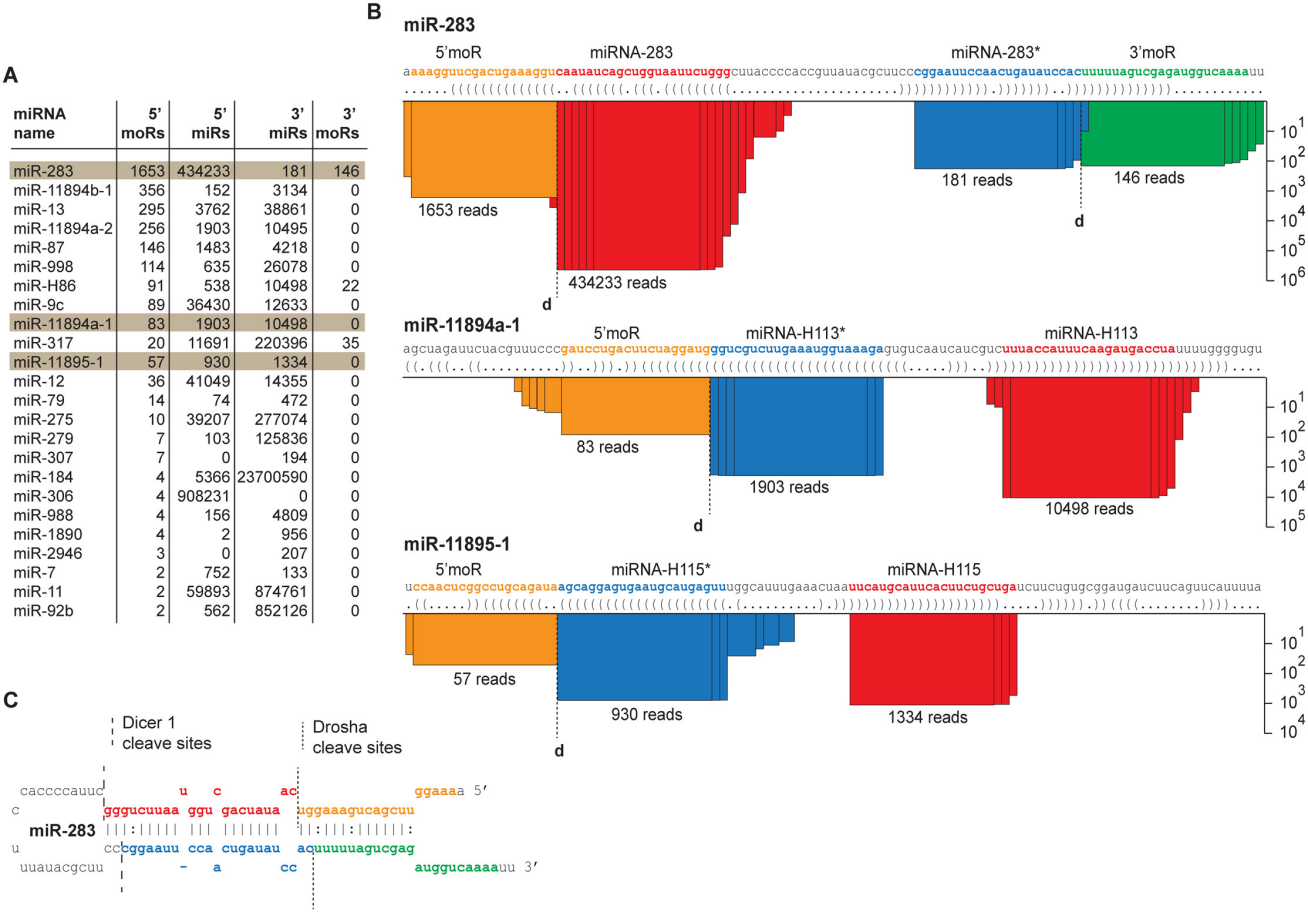
miRNA offset RNAs in Aag2 cells. **(A)** List of miRNA hairpins that give rise to moRs (sorted by the total moR count). The combined miRNA and moR read count from all six deep-sequencing libraries is shown. Highlighted miRNAs are described in more detail in panel B. **(B)** Three examples of mature miRNAs and moRs mapping to miRNA hairpins. The height of the bar (on log scale) reflects the number of reads covering the corresponding nucleotide position. The total amount of miRNA/moR reads is indicated below each bar stack. The most abundant miRNA/moR sequence is highlighted using the following color coding: orange, 5’ moR; red, mature miRNA; blue, miRNA*; green, 3’ moR. The dashed vertical line marked with a ‘d’ reflects the putative Drosha cleavage site. **(C)** miR-283 hairpin with 5’ and 3’ miRNA/moR sequences highlighted with colored nucleotide letters (see panel B). The sites of Dcr1 and Drosha cleavage are indicated by the dashed lines.

During the analysis of miRNA predictions, we noted the expression of specific small RNAs adjacent to the mature miRNA and miRNA* star strands. These miRNA offset RNAs (moRs) have been detected in small RNA deep-sequencing data from invertebrates, simple chordates, vertebrates and even viruses [61–68]. In total, we identified moRs for 27% (24/89) of previously reported miRNA hairpins. In many of these cases (nine out of 24), the number of moRs per hairpin is below one thousandth of the number of mature miRNA reads. Others were more abundant, with moR reads accumulating up to 3.6% of the number of mature miRNAs. In a single instance, miR-11894b-1, the number of moRs reached 10.8% of the number of mature miRNA reads. In agreement with previous findings in *Drosophila* [63] 5’ moRs were more abundant than 3’ moRs (Fig 6A). moRs have been proposed to be the by-products of Drosha cleavage and in line with this suggestion, we found the 3’ end of 5’ moRs and the 5’ end of 3’ moRs to be fixed, reflecting potential Drosha cleavage sites (Fig 6B and 6C). In contrast, the ends of moRs facing the termini of the miRNA stemloop were less well defined, suggesting that they are processed by exonuclease activity (Fig 6B). Why certain miRNA hairpins are prone to accumulation of moRs remains unclear.

## Discussion

Small RNA pathways critically influence the outcome of virus infections in many host organisms, including plants, fungi, invertebrates, and vertebrates [3–5,15,69]. In plants and invertebrates, siRNA-mediated antiviral immunity is key to the defence against a broad range of virus infections. In *Aedes* mosquitoes, viral siRNAs were detected from several virus families, including *Togaviridae*, *Flaviviridae*, *Bunyaviridae*, and *Reoviridae* [5]. In line with previous reports, our analysis identified viral siRNAs derived from the entire genomic RNA of DENV2 in *Aedes aegypti* cells [35,36]. These vsiRNAs are produced in roughly equal amounts from the (+) strand and the (-) strand of the virus, indicating that the dsRNA replication intermediates serve as substrate for Dcr2. Upon knockdown of either Dcr2 or Ago2 in whole *Aedes* mosquitoes, DENV2 titres and transmission are enhanced, underlining the pivotal role of RNA interference in limiting DENV2 replication [53].

Besides siRNAs, our DENV2 infected small RNA libraries contained a substantial number of virus-mapping reads in the size range of piRNAs. Their expression was confirmed using small RNA northern blotting, validating that these small RNA reads were no sequencing artefacts. Only very few DENV2 genomic locations near the 3’ end of the DENV genome give rise to these vpiRNAs but the origin of this spiky pattern remains obscure. We hypothesized that perhaps endogenous, transposon-derived piRNAs would loosely bind the DENV2 genome at these positions triggering the production of secondary vpiRNAs. A similar mechanism has been suggested to initiate piRNA production from specific mRNAs in *Drosophila* [28]. However, various mapping strategies allowing small RNA alignment with up to six mismatches did not uncover endogenous piRNAs that could trigger vpiRNA production at the observed positions.

Previous analyses of DENV2-derived small RNAs identified vpiRNAs in *Aedes aegypti* mosquitoes or Aag2 cells [35,36]. Also in the viral small RNA population reported by Scott and colleagues [35], a major small RNA spike is located near the 3’ end of the DENV genome. Since the entire population of viral RNA is analyzed in this study, it is however hard to assess which type of small RNA contributes to the spike. Interestingly, the spiky genome distribution of vpiRNAs is also recapitulated in adult *Aedes* mosquitoes. Although in this study the exact location of vpiRNA spikes differs from the positions we found in Aag2 cells, these data suggest that similar mechanisms may be responsible for piRNA biogenesis in Aag2 cells and adult mosquitoes. Yet, genetic evidence for the PIWI protein dependency of vpiRNA production *in vivo* is lacking. Of note, piRNAs are far less abundant in the small RNA libraries reported by Scott *et al*. [35] and Hess *et al*. [36] compared to our data from Aag2 cells. These differences might be due to different experimental conditions, including the chosen MOI, the time point of sampling, or to differences in small RNA library preparation and sequencing methodology. In addition, the specific viral strains may critically influence the accumulation of vpiRNAs. We have tested two laboratory-adapted DENV2 strains which both give rise to vpiRNAs, and it would be interesting to test if pathogenic strains from DENV2 endemic areas would show similar phenotypes.

Using knockdown of PIWI proteins, we identified Piwi5, Ago3 and, to a lesser extent, Piwi6 as responsible for the production of vpiRNAs in Aag2 cells. Therefore, DENV2 vpiRNA biogenesis in Aag2 cells relies on a similar set of PIWI proteins as SINV vpiRNAs, which also depend on Piwi5 and Ago3 [37]. We have recently proposed that *Aedes* PIWI proteins are specialized in producing piRNAs from various sources. Whereas in mosquito cells piRNA biogenesis from transposons directly and indirectly depends on Piwi4-6 and Ago3, piRNA biogenesis from SINV predominantly requires Piwi5 and Ago3 only [37]. The additional involvement of Piwi6 for piRNA production from DENV2 suggests that *Aedes* PIWI proteins are even further specialized towards RNA substrates from different viruses. This may be caused by virus-specific sequence elements or structures that are preferentially recognized by certain PIWI proteins. Alternatively, but not mutually exclusive, differences in replication strategies or replication sites might favour recognition of viral RNA by distinct sets of PIWI proteins.

The almost complete loss of DENV2 piRNAs upon knockdown of Piwi5 and Ago3 indicates that both proteins are equally important for vpiRNA biogenesis in Aag2 cells, similar to piRNA biogenesis during SINV infection [37]. SINV piRNAs are produced by a two-step amplification mechanism that resembles ping-pong amplification of transposon piRNAs in *Drosophila* [27,29]. During this process, a piRNA-loaded PIWI protein (Piwi5 in *Aedes* or Aubergine in *Drosophila*) slices a complementary target RNA and transfers the 3’ slicer products as the new piRNA precursor to a second PIWI protein (Ago3 in *Aedes* and *Drosophila*). From this precursor, an Ago3-bound secondary piRNA is produced that in turn is able to slice a target RNA, giving rise to a new piRNA precursor. This precursor will be matured to generate the same primary piRNA sequence that initiated the amplification. Therefore, this model predicts the presence of piRNAs derived from both strands. Although also during SINV infection (-) strand derived piRNAs are only a minor fraction, they can be identified as the primary piRNAs by a nucleotide bias that is characteristic for Piwi5/Aub bound piRNAs (uridine at position one). During DENV2 infection, 25–30 nt reads from the (-) strand are extremely scarce and they do not have the nucleotide bias that would classify them as primary piRNAs. Therefore, exactly how the production of the secondary, (+) strand vpiRNAs is triggered or whether a different, amplification-independent mechanism is responsible for their production, remains unclear.

The expression of miRNAs from DENV2 genomic RNA is still debated. Based on small RNA sequencing data, Hussain and Asgari have described a set of six viral small RNAs that have miRNA-like properties [24]. Inhibition of one of them, vsRNA-5, by complementary RNA molecules strongly enhances DENV virus replication [24]. However, expression of these viral small RNAs is generally not high and the relevance of such a lowly abundant small RNA during the exponential growth of a virus was therefore questioned [25]. In our dataset, we find a high number of specific vsRNA reads for only vsRNA-2. For all the other predicted vsRNAs we find no or very low numbers of reads. vsRNA-2 is located on a hairpin at the very end of the DENV2 genome. This strongly resembles KUN-miRNA1, a viral small RNA expressed in mosquito cells infected with West Nile virus, a related flavivirus [70]. KUN-miR1 is 21nt in size and its expression is Dcr1-dependent. In contrast, our data demonstrate that vsRNA-2 has a broad size distribution of primarily 26–28 nt, arguing against it being a canonical Dicer-dependent miRNA. Altogether, these data support the notion that miRNA-like small RNAs from DENV2 are extremely lowly abundant, with a questionable role in the regulation of viral replication.

Modulation of host miRNAs after virus infection may be a mechanism that coordinates gene expression during the course of the immune response. Alternatively, it may be a consequence of a viral strategy to manipulate host gene expression. Comparing uninfected with DENV2-infected Aag2 cells showed that the expression of almost all miRNAs was unchanged upon infection. Only a handful of miRNAs were up or down-regulated after exposure to DENV2. The fold changes ranged from approximately 4 fold up to 4 fold down. In whole *Aedes aegypti* mosquitoes, a total of 31 miRNAs were recently shown to be differentially regulated following infection at three different time points [17]. The set of differentially expressed miRNAs is inconsistent between the different analyzed time points (2, 4 and 9 days post infection, dpi), but the number of differentially expressed miRNAs was higher at nine dpi than at two or four dpi. These data suggest that changes in miRNA expression may be more prominent in a long-term infection and we thus cannot exclude the possibility that prolonged infection of Aag2 cells may result in more pronounced changes in miRNA expression. Alternatively, the observed miRNA changes in adult mosquitoes might not directly happen in infected cells *per se*, but could reflect an indirect effect of homeostatic or metabolic responses during the infection. It should be remembered that miRNA expression can be highly cell-type specific; if miRNA levels are responsive to DENV2-infection only in selected cell types within the entire mosquito, we may miss those in our Aag2 cell-based assays.

In summary, here we provide an in-depth analysis of small RNAs in DENV2 infected Aag2 cells in comparison to uninfected cells. Aag2 cells provide a powerful model system for studying biochemical details of small RNA biogenesis pathways, as they are fully competent in producing siRNAs, miRNAs and piRNAs originating from both virus and host. Our analyses add both DENV-derived piRNAs and novel *Aedes aegypti* miRNAs to the small RNA repertoire in this medically important virus-host interaction.

## Supporting Information

S1 Fig DENV small RNAs in *Aedes* mosquitoes. Re-analysis of small RNA sequencing data from DENV2-JAM1409 infected *Aedes* mosquitoes (9 days post infection) published by Hess *et al*. [36]. **(A)** Size profile of small RNA reads mapping to the sense strand (black) or antisense strand (grey) of the DENV2 JAM1409 genome. Inlay shows reads of 23 to 32 nt in size, with a different scale for the y-axis. **(B)** Peaks indicate the number of 5’ends of small RNAs of 21 nt (left panel) or 25–30 nt (right panel) across the sense (red) or antisense (blue) strand of the viral genome. Read counts have been normalized to the depth of the library. Red numbers in D and E indicate the genome position of the 5’ end of the small RNA peaks.
(TIF)

S2 Fig Effect of PIWI knockdown on DENV2 RNA levels. **(A)** Quantification of DENV2 RNA levels by RT-qPCR in the samples used for Fig 2E and 2F. **(B)** Knockdown (KD) efficiency of Piwi5 and Ago2 (upper panel) and the corresponding levels of DENV2 RNA (lower panel) as assessed by RT-qPCR. Bars indicate mean +/- SEM of three independent experiments. Statistical significance was determined using two tailed, unpaired student t-test. * p<0.05; **p<0.01.
(TIF)

S3 Fig DENV miRNAs are not expressed in Aag2 cells. **(A)** miRNA predictions using miRDeep2 on the DENV2-NGC genome. The read counts of the individual sequences, combined from all the six sequencing libraries, that support the predictions are shown to the left. The predicted mature miRNA and the expected miRNA* sequences are indicated in red and blue, respectively. **(B)** Genome coverage of small RNAs mapping to the DENV2-NGC 3’ UTR. Bars represent the mean of the normalized coverage at each nucleotide position (n = 3). Green bars show all small RNA reads mapping to the region. Red bars show the 22–24 nt reads derived from the viral (+) strand only. **(C)** Size distribution of (+) strand-derived small RNA reads that end at the 3’ terminus of the DENV2-NGC genome. Bars represent the average read count and SEM of the three deep-sequencing libraries normalized to their corresponding size (displayed as % of library).
(TIF)

S4 Fig New *Aedes* miRNAs reported by Hu *et al*. or Akbari *et al*. Folding of miRNA hairpins that have been published by Hu *et al*. or Akbari *et al*. [57,58], and have also been recovered by the miRDeep2 analyses of our libraries. These miRNAs were not yet published in the most recent version of miRBase. The folding of the hairpin was predicted using RNAfold. Red and blue letters indicate the position of the predicted, mature miRNA and miRNA* sequences, respectively. The shown miRNA names have been assigned by miRBase; the previous names given by the authors of the indicated studies are presented between brackets. The mature miRNA sequences of aae-miR-11893-1 maps to similar hairpins at two locations in the *Aedes* genome. Also aae-miR-11894a-1 and aae-miR-11894a-2 are identical sequences that map to two very similar hairpins. Aae-miR-11895-1 and aae-miR-11895-2 are two identical hairpins that map to two locations in the *Aedes* genome. Similarly, the mature aae-miR-11899 sequence maps to two identical hairpins in the genome.
(TIF)

S1 Table Name and sequence of oligonucleotides used in this study. (PDF)

S2 Table Spreadsheet of raw miRDeep2 output. Sheet 1 provides an overview of the selection procedure to reach the final miRNA predictions. Sheets 2 to 6 show the unfiltered miRNA predictions and the filtered miRNA predictions after the individual rounds of selection.
(XLSX)

S1 Dataset Data related to Figs 1C, 1D, 2E, 2F, 3, 4, S1, S2, S3B and S3C. (XLSX)
