# Supplementary figures and images for: Small RNA Profiling in Dengue Virus 2-Infected Aedes Mosquito Cells Reveals Viral piRNAs and Novel Host miRNAs
Source: PLoS Negl Trop Dis. 2016 Feb 25;10(2):e0004452. doi: 10.1371/journal.pntd.0004452 (PMC4767436; doi:10.1371/journal.pntd.0004452)

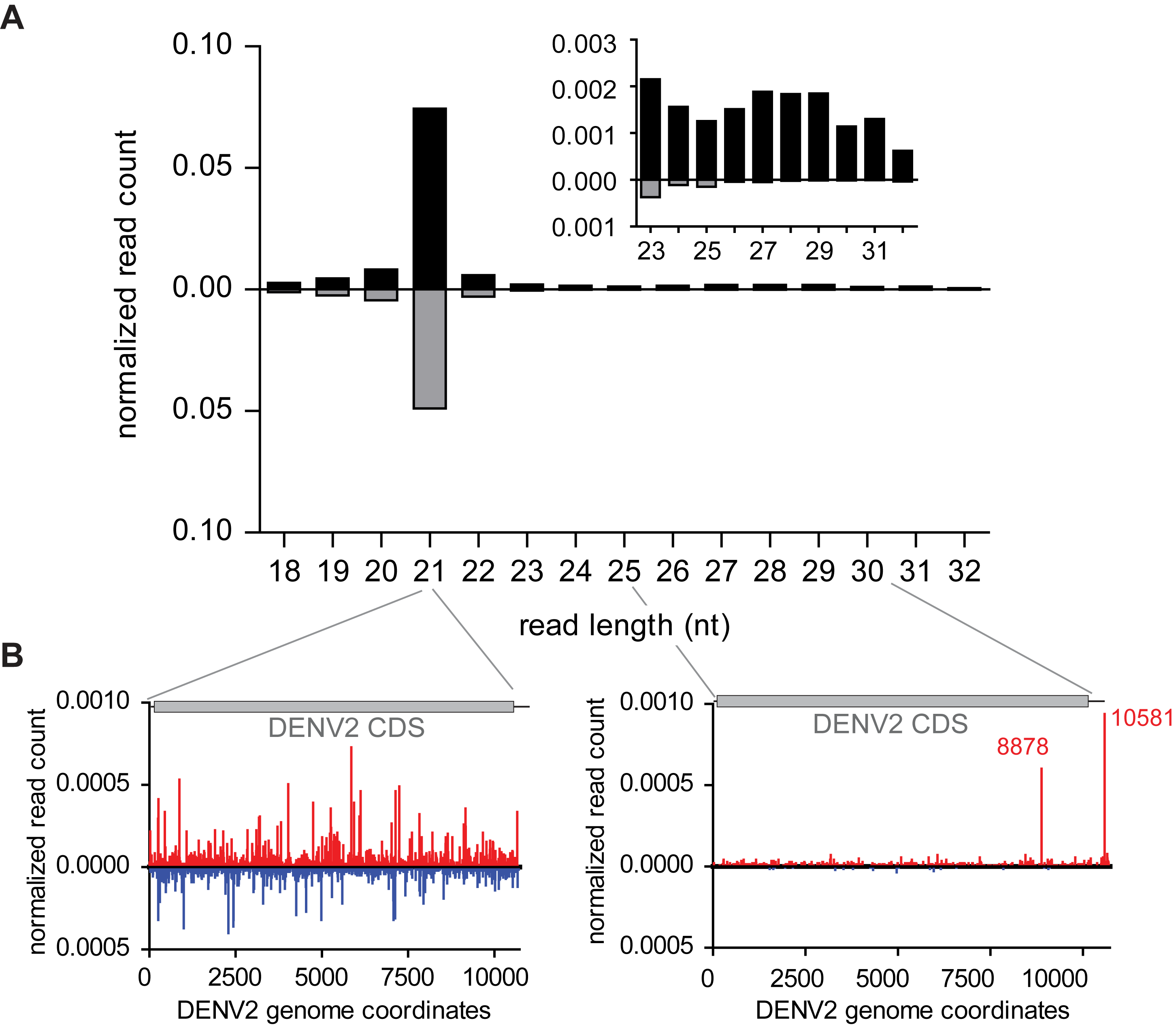

Supplement: S1 Fig — Re-analysis of small RNA sequencing data from DENV2-JAM1409 infected Aedes mosquitoes (9 days post infection) published by Hess et al. [36]. (A) Size profile of small RNA reads mapping to the sense strand (black) or antisense strand (grey) of the DENV2 JAM1409 genome. Inlay shows reads of 23 to 32 nt in size, with a different scale for the y-axis. (B) Peaks indicate the number of 5’ends of small RNAs of 21 nt (left panel) or 25–30 nt (right panel) across the sense (red) or antisense (blue) strand of the viral genome. Read counts have been normalized to the depth of the library. Red numbers in D and E indicate the genome position of the 5’ end of the small RNA peaks. (TIF) [file pntd.0004452.s001.tif]

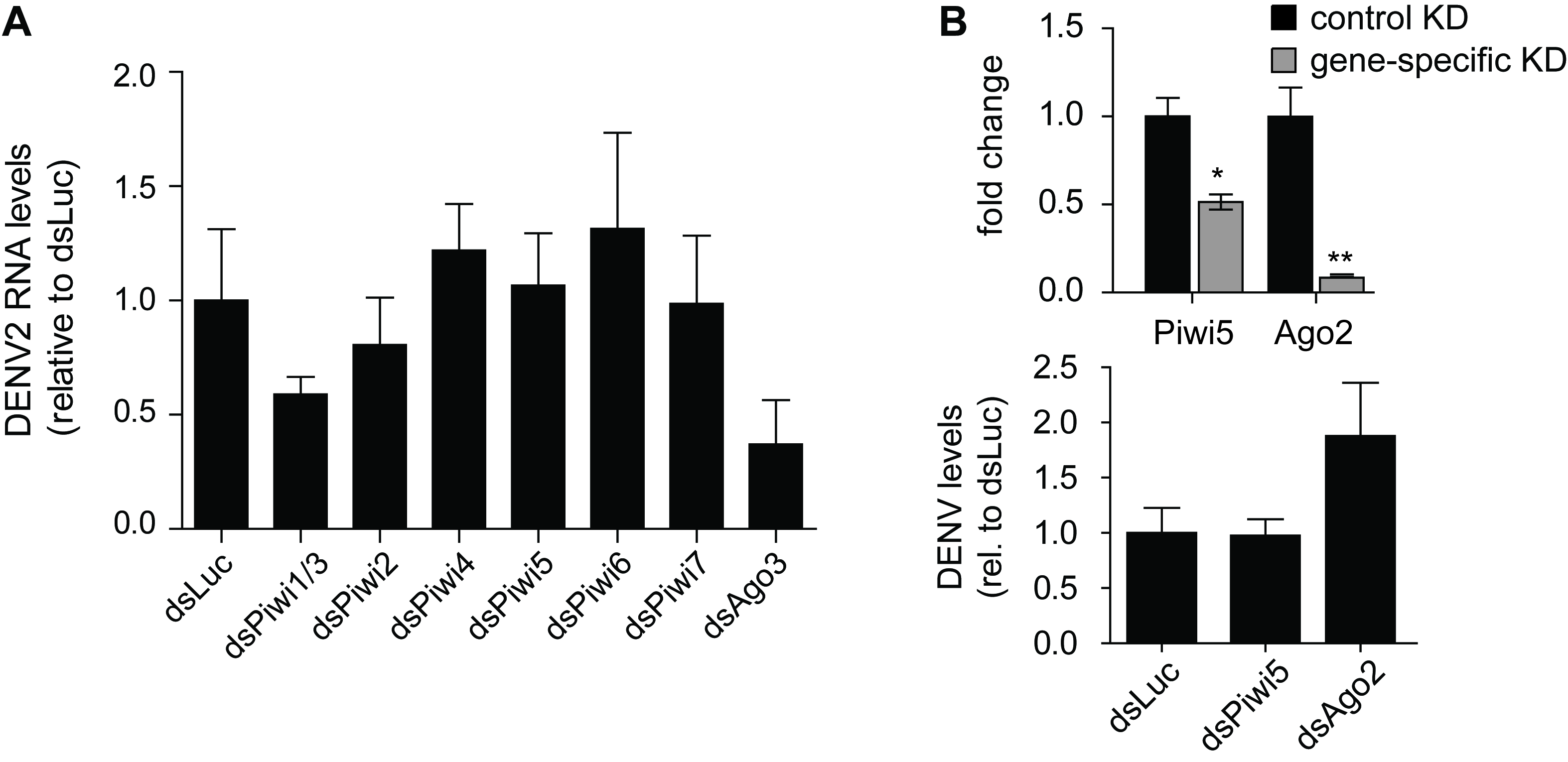

Supplement: S2 Fig — (A) Quantification of DENV2 RNA levels by RT-qPCR in the samples used for Fig 2E and 2F. (B) Knockdown (KD) efficiency of Piwi5 and Ago2 (upper panel) and the corresponding levels of DENV2 RNA (lower panel) as assessed by RT-qPCR. Bars indicate mean +/- SEM of three independent experiments. Statistical significance was determined using two tailed, unpaired student t-test. * p<0.05; **p<0.01. (TIF) [file pntd.0004452.s002.tif]

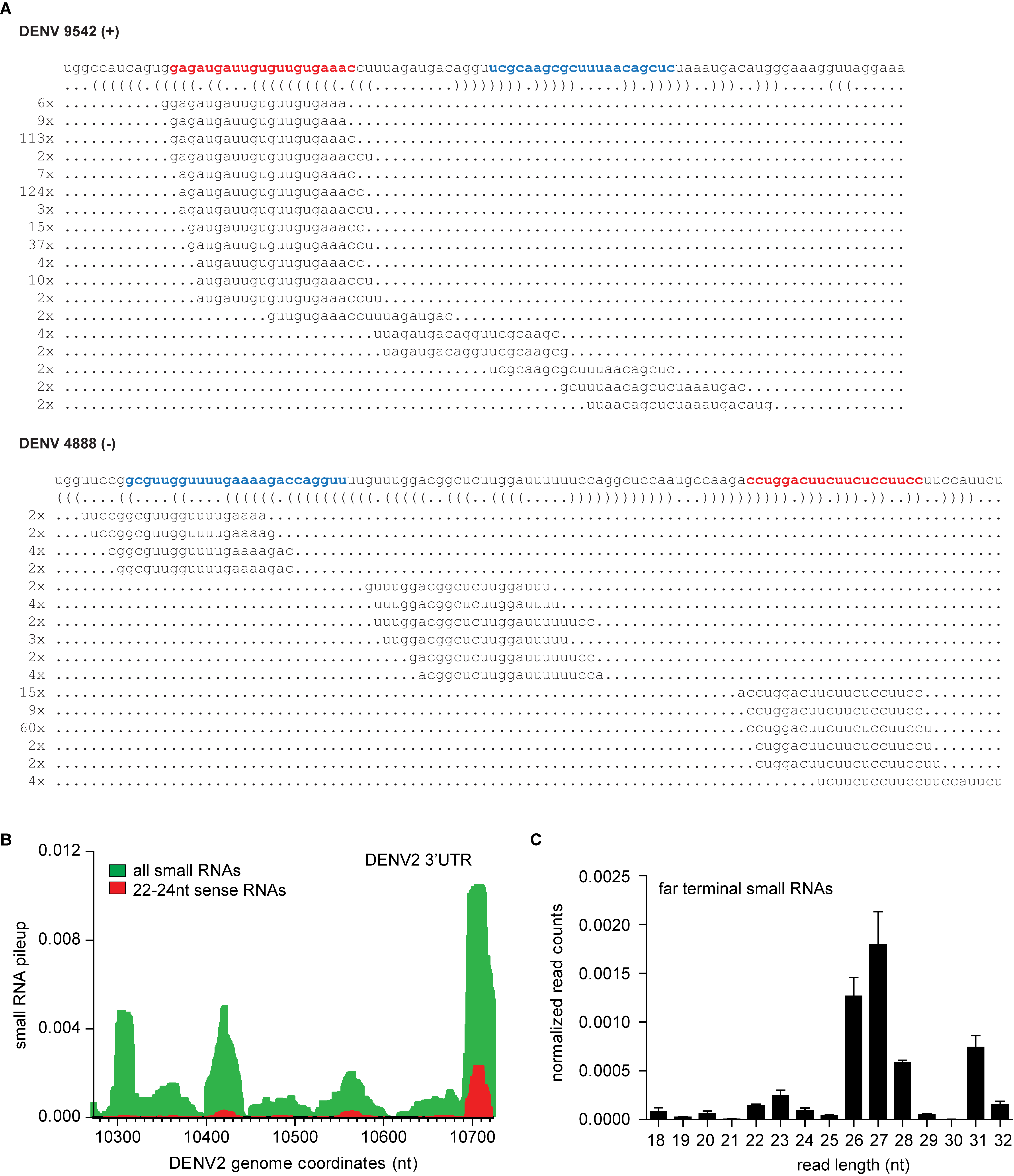

Supplement: S3 Fig — (A) miRNA predictions using miRDeep2 on the DENV2-NGC genome. The read counts of the individual sequences, combined from all the six sequencing libraries, that support the predictions are shown to the left. The predicted mature miRNA and the expected miRNA* sequences are indicated in red and blue, respectively. (B) Genome coverage of small RNAs mapping to the DENV2-NGC 3’ UTR. Bars represent the mean of the normalized coverage at each nucleotide position (n = 3). Green bars show all small RNA reads mapping to the region. Red bars show the 22–24 nt reads derived from the viral (+) strand only. (C) Size distribution of (+) strand-derived small RNA reads that end at the 3’ terminus of the DENV2-NGC genome. Bars represent the average read count and SEM of the three deep-sequencing libraries normalized to their corresponding size (displayed as % of library). (TIF) [file pntd.0004452.s003.tif]

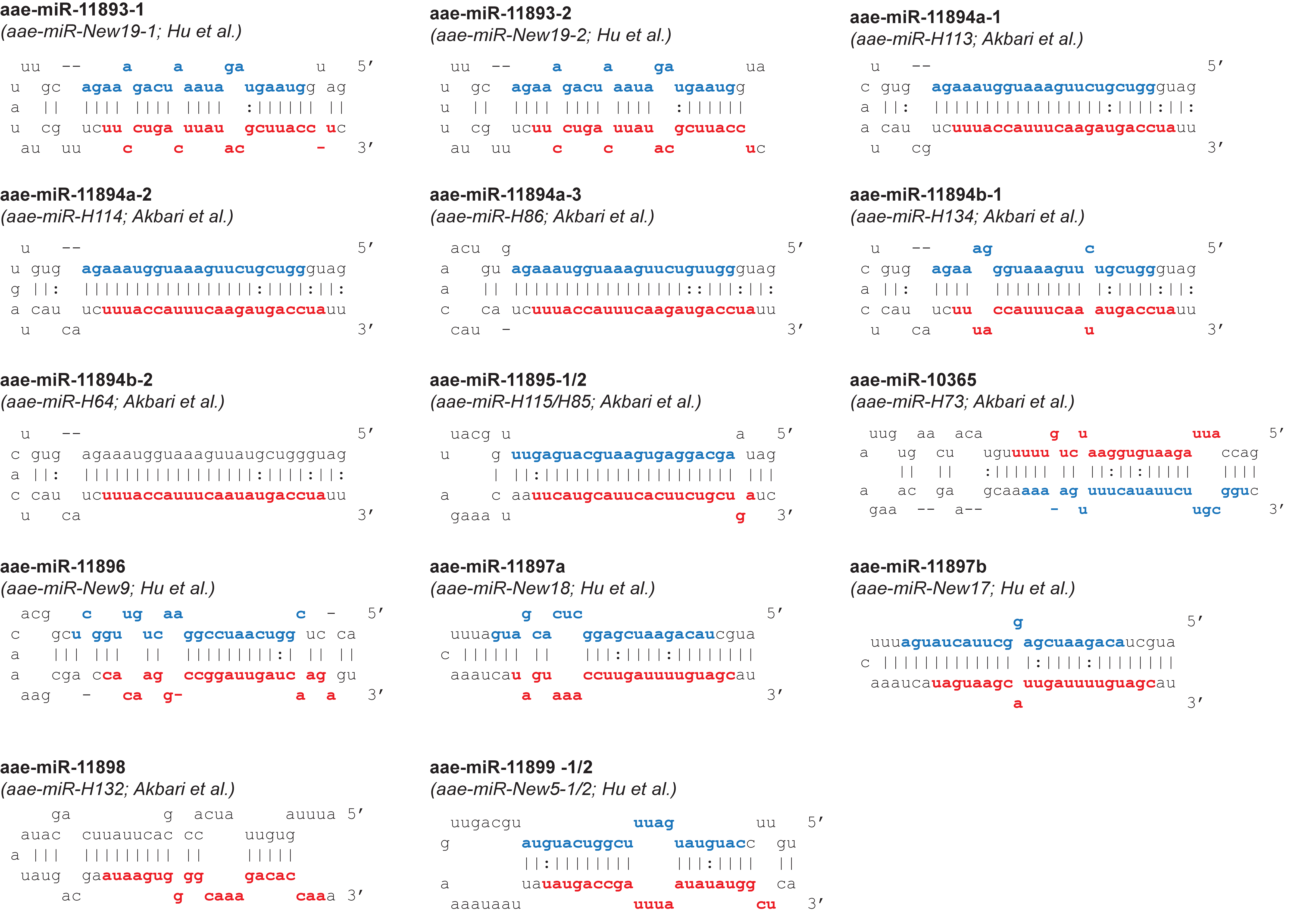

Supplement: S4 Fig — Folding of miRNA hairpins that have been published by Hu et al. or Akbari et al. [57,58], and have also been recovered by the miRDeep2 analyses of our libraries. These miRNAs were not yet published in the most recent version of miRBase. The folding of the hairpin was predicted using RNAfold. Red and blue letters indicate the position of the predicted, mature miRNA and miRNA* sequences, respectively. The shown miRNA names have been assigned by miRBase; the previous names given by the authors of the indicated studies are presented between brackets. The mature miRNA sequences of aae-miR-11893-1 maps to similar hairpins at two locations in the Aedes genome. Also aae-miR-11894a-1 and aae-miR-11894a-2 are identical sequences that map to two very similar hairpins. Aae-miR-11895-1 and aae-miR-11895-2 are two identical hairpins that map to two locations in the Aedes genome. Similarly, the mature aae-miR-11899 sequence maps to two identical hairpins in the genome. (TIF) [file pntd.0004452.s004.tif]
