# Supplementary material for: Small RNA Profiling in Dengue Virus 2-Infected Aedes Mosquito Cells Reveals Viral piRNAs and Novel Host miRNAs
Source: PLoS Negl Trop Dis. 2016 Feb 25;10(2):e0004452. doi: 10.1371/journal.pntd.0004452 (PMC4767436; doi:10.1371/journal.pntd.0004452)
Supplement: S1 Table — (PDF) [file pntd.0004452.s005.pdf]

S1 Table: Oligonucleotides used in this study

**Name**	**Sequence**
Primers used for the production of T7-flanked PCR products	
T7F-Luc	taatacgactcactatagggagaTATGAAGAGATACGCCCTGGTT
T7R-Luc	taatacgactcactatagggagaTAAAACCGGGAGGTAGATGAGA
T7F-Piwi1/3	taatacgactcactatagggagaCCACGCCCATCGTTTCAA
T7R-Piwi1/3	taatacgactcactatagggagaCCTCAGTTTGTTCACCATA
T7F-Piwi2	taatacgactcactatagggagaCCGTCCTACTTTCCAGCAC
T7R-Piwi2	taatacgactcactatagggagaGCGGCACTCCAGGGACAAT
T7F-Piwi4	taatacgactcactatagggagaCGTGGAAGTCCTTCTTCTCG
T7R-Piwi4	taatacgactcactatagggagaTGTCAGTTGATCGCTTCTCAA
T7F-Piwi5	taatacgactcactatagggagaGCCATACATCGGGTCAAAAT
T7R-Piwi5	taatacgactcactatagggagaCTCTCCACCGAAGGATTGAA
T7F-Piwi6	taatacgactcactatagggagaCAACGGAGGATCTTCACGAG
T7R-Piwi6	taatacgactcactatagggagaAATCGATGGCTTGATTTGGA
T7F-Piwi7	taatacgactcactatagggagaGTGGAGGTCGTGGAGGTAAC
T7R-Piwi7	taatacgactcactatagggagaGTTTGCGGTGTTTCCGTACT
T7F-Ago3	taatacgactcactatagggagaTGCTTACTCGTGTCGCGTAG
T7R-Ago3	taatacgactcactatagggagaGGCATGGCAGATCCAATACT
(*quantitative) PCR primers*	
F-Piwi4	TCTTCTTCTCCACCACAGCC
R-Piwi4	ATGGTGACCACCTCACAGTTAC
F-Piwi5	ACGGCATCACATCGAGACTC
R-Piwi5	CGACCTCCACGCTGTCCTC
F-Piwi6	TTTTCTTCCACCCCGAGCAG
R-Piwi6	AATACATTTGCGATGCGGCC
F-Ago3	CTCCAGACGACGGTTTTGGA
R-Ago3	GCAGGTACGAAATTGGCTGC
F-Ago2	ATTTGGCTCAAGATCAACGC
R-Ago2	GAGATCGTATGAAGCGGCCA
F-LAP	GTGCTCATTCACCAACATCG
R-LAP	AACTTGGCCGCAACAAATAC
F-DV2-NS1	AGAACTGAAGTGTGGCAGTGGGAT
R-DV2-NS1	TGCCCTCTTCATGAGCTTTCTGGA
Northern blot probes	
nDV2-9180+	GGTCTTCTAGTGTGATTCTTGTGTCCCAT
nDV2-9985+	CCCTGTTCCAGACTGTCAGCATGTCTTCCGT
nMiR-2940-3p	AGTGATTTATCTCCCTGTCGAC
